# A survey on computer aided diagnosis for ocular diseases

**DOI:** 10.1186/1472-6947-14-80

**Published:** 2014-08-31

**Authors:** Zhuo Zhang, Ruchir Srivastava, Huiying Liu, Xiangyu Chen, Lixin Duan, Damon Wing Kee Wong, Chee Keong Kwoh, Tien Yin Wong, Jiang Liu

**Affiliations:** 1 Institute for Infocomm Research, 1 Fusionopolis Way, Singapore, Singapore; 2 Nanyang Technological University, Nanyang Drive, Singapore, Singapore; 3 Singapore National Eye Centre, Third Hospital Avenue, Singapore, Singapore

**Keywords:** Computer Aided Diagnosis (CAD), Ocular diseases, Review, Clinical data, Ocular imaging, Genetic information

## Abstract

**Background:**

Computer Aided Diagnosis (CAD), which can automate the detection process for ocular diseases, has attracted extensive attention from clinicians and researchers alike. It not only alleviates the burden on the clinicians by providing objective opinion with valuable insights, but also offers early detection and easy access for patients.

**Method:**

We review ocular CAD methodologies for various data types. For each data type, we investigate the databases and the algorithms to detect different ocular diseases. Their advantages and shortcomings are analyzed and discussed.

**Result:**

We have studied three types of data (i.e., clinical, genetic and imaging) that have been commonly used in existing methods for CAD. The recent developments in methods used in CAD of ocular diseases (such as Diabetic Retinopathy, Glaucoma, Age-related Macular Degeneration and Pathological Myopia) are investigated and summarized comprehensively.

**Conclusion:**

While CAD for ocular diseases has shown considerable progress over the past years, the clinical importance of fully automatic CAD systems which are able to embed clinical knowledge and integrate heterogeneous data sources still show great potential for future breakthrough.

## Background

Patients with ocular diseases are often unaware of the asymptomatic progression of the said disease [1] until at a later stage when treatment is less effective in preventing vision impairment [2]. Though regular eye screenings enable early detection and timely intervention of such diseases, it would put a significant strain on limited clinical resources. Computer Aided Diagnosis (CAD) systems, which automate the process of ocular disease detection, are urgently needed to alleviate the burden on the clinicians.

Owing to the fast pace of technological advancements in both hardware and software, many CAD systems have been developed for the diagnosis of ocular diseases over the past years, though most of them are still undergoing evaluation or clinical validation. For example, Fujita et al. [3] discussed an emerging CAD system using retinal fundus images for the detection of glaucoma, diabetic retinopathy (DR) and hypertensive retinopathy. Their project has entered the final stage of development, and commercialized CAD systems ought to appear by its completion.

Though such fully automated systems are not yet on the market, semi-automated and manual computer systems incorporating these CAD systems are relatively widely used, with several clinical publications already reporting on their usage. Examples of the development of such systems include IVAN [4] from University of Wisconsin and more recently SIVA from National University of Singapore [5] for semi-automated vascular analysis. Software packages allowing for processing of data garnered from these systems also exist: ADRES 3.0 by Perumalsamy et al. [6] is used for the grading of DR and has been commercialised and deployed for use in diabetic centres and general physician clinics in India; the Singapore Eye Research Institute has also been running clinical trials for the diagnosis of several ocular diseases (e.g., pathological myopia (PM), DR and age related macular degeneration (AMD)) using a uniform set of ophthalmic image reading and analysis protocols [7].

This survey covers three types of data for CAD systems: clinical data, image based data and genetic data. Clinical data refers to a patient’s demographic information (e.g., age, race etc.) and data acquired from clinical laboratory tests or exams, e.g. intra-ocular pressure (IOP), but excludes data acquired from digital imaging or genomic tests (Section “Result: CAD of ocular diseases based on clinical data”). Image based data refers to images captured using an imaging device for observing the pathology in the affected part of the eye (details are in Section “Imaging modalities”). Genetic information refers to any data obtained from an individual’s DNA, genes or proteins (Section “Result: predicting ocular diseases based on genetic information”). These definitions are specific to this paper and may vary depending on context. Of the three data types, CAD systems using clinical data has already been widely studied in the clinical field [8-10]. As far as CAD using genetic information is concerned, recent advancements in genotyping technology have made individual genetic information more commonly available, but it is still unfeasible to utilise genetic information for CAD systems on a large scale presently. Perhaps with time, genetic information will find its rightful place in medicine by supplementing phenotypic clinical data with validated genetic interpretations [11]. We cover genetic data as a possible input to future CAD systems. A considerable amount of the survey is focused on the usage of image based data in CAD systems as they are by far the most important type of data in ocular disease diagnosis.

There have been surveys on retinal imaging in the area of ocular research [12,13]. However, there lacks a broader literature survey on using CAD for ocular disease diagnosis. This has motivated us to write a systematic review of recently developed methods for CAD in ocular research.

## Methods

In this work, we review research and development on automatic ocular disease diagnosis in the light of three data types, viz. clinical, image and genetic. For each data type, we investigate the algorithms and available databases developed for different ocular diseases. The associated publications were retrieved from two literature databases, PubMed and IEEEXplore. Considering the works which use images as data, to understand the major image modalities used for CAD applications and the trends of research areas, we summarize the statistics of image-based studies conducted on various ocular diseases. We examine the biomedical databases to extract the known genetic information regarding ocular diseases.

The results of the review are presented in three sections: Sections “Result: CAD of ocular diseases based on clinical data” and “Result: CAD of ocular diseases based on imaging” describe the CAD of ocular diseases based on clinical data and ocular imaging respectively. Section “Result: predicting ocular diseases based on genetic information” concerns studies relating genomic informatics to disease prediction. Furthermore, in Section “Discussion” we discuss the observed trends in the field and the possibility of CAD systems based on integrated data sources.

## Result: CAD of ocular diseases based on clinical data

One of the pioneer research works on Clinical Decision Support Systems (CDSS), CASNET [14] (causal-associational network), was developed in late 1970s to assist in the diagnosis of glaucoma. Clinical data used in CASNET covered symptoms reported by the patient, e.g., ‘ocular pain’, ‘decreased visual acuity’ and various eye examination results, e.g. visual acuity, IOP, anterior chamber depth, angle closure, pupil abnormality and corneal edema [15]. CASNET used a descriptive model of the disease process for logical interpretations of clinical findings for glaucoma. The model representing pathophysiological mechanisms had the form of a semantic net with weighted links. It represented early medical expert systems, providing a framework describing the knowledge of expert consultants and simulating various aspects of the cognitive process of clinicians.

In 2002, Chan et al. [16] reported the first implementation of Support Vector Machines (SVM) in glaucoma diagnosis. Clinical data used in the research was the output from Standard Automated Perimetry (SAP), a common computerized visual field test. The authors compared the performance of a number of machine learning algorithms with SAP output. The machine learning algorithms studied included multilayer perceptron (MLP), SVM, Linear and Quadratic Discriminant Analysis (LDA and QDA), Parzen window, mixture of Gaussian (MOG), and mixture of generalized Gaussian (MGG). It was observed that machine-learning-type classifiers showed improved performance over the best indexes from SAP. The authors also discussed the advantage of using feature selection to further improve the classification accuracy with a potential to reduce testing time by diminishing the number of visual field location measurements.

In 2011, Bizios et al. [17] conducted a study investigating the data fusion methods and techniques for simple combinations of parameters obtained from SAP and measurements of the Retina Nerve Fibre Layer Thickness (RNFLT) obtained from Optical Coherence Tomography (OCT) for diagnosis of glaucoma using Artificial Neural Networks. The results showed that the diagnostic accuracy from a combination of fused SAP and OCT data was higher than using either of the two alone. This was the first reported study using fused data for glaucoma diagnosis.

A recent study [18] investigates the relationship between the central corneal thickness (CCT), Heidelberg Retina Tomography II (HRTII) structural measurements and IOP using an innovative non-linear multivariable regression method, in order to define the risk factors in future glaucoma development.

Two recent works on ocular disease diagnosis based on clinical data need to be mentioned here. Liu et al. [19] developed an automatic glaucoma diagnosis and screening architecture, automatic glaucoma diagnosis through medical imaging informatics (AGLAIA-MII), which combined subjects’ personal data, imaging information from Digital Fundus Photographs (DFPs), and patients’ genome information for glaucoma diagnosis. Features from each data source were extracted automatically. Subsequently, these features were passed to a multiple kernel learning (MKL) framework to generate a final diagnosis outcome. In another work, Zhang et al. [20] proposed a computer-aided diagnosis framework for Pathological Myopia (PM) based on Biomedical and Image Informatics. These heterogeneous data sources contained fundus images, demographic/clinical and genetic data. Their system combined these potentially complementary pieces of information to enhance the understanding of the disease, providing a holistic appreciation of the multiple risks factors as well as improving the diagnostic outcomes. A data-driven approach was proposed to exploit the growth of heterogeneous data sources to improve assessment outcomes.

Other less prevalent diseases which are detected using clinical data are briefly explained in the following:

### 

#### 

##### 

**Trachoma:** Most people with trachoma in its initial stages display no signs or symptoms. Clinically the diagnosis of trachoma can be done by using magnifiers and a flashlight (physical examination) or through a cultural sample of bacteria from the eye tested in a laboratory [21].

##### 

**Onchocerciasis:** Onchocerciasis is the 2nd leading cause of infectious blindness worldwide. Also called ‘river blindness’, it is a skin and eye disease caused by the parasitic worm and spread by blackflies that breed in fast-flowing water. The two common diagnostic techniques are skin biopsies and serological assays [22].

### Clinical databases

There are a number of large scale or population-based eye studies conducted in various countries. For example, 

• Blue Mountains Eye Study (Australia) [23]

• Singapore Malay Eye Study [24]

• Singapore Indian Eye Study [25]

• Singapore Chinese Eye Study [26]

Many research works conducted on various ocular diseases have been published based on the data collected in these eye studies. However, the data is not publicly available in research community.

## Result: CAD of ocular diseases based on imaging

In ophthalmology, ocular imaging has developed rapidly during the past 100 over years and play an critical role in clinical care and ocular disease management [27]. Large-scale systematic research and development of CAD from radiology and medical images began in the early 1980s. The first report on retinal image analysis was published in 1973, focusing on vessel segmentation [28]. In 1984, Baudoin et al. [29] described an image analysis method for detecting lesions related to DR.

Over the past 20 years, developments in image processing relevant to ophthalmology have paved the way for the development of automated diagnostic systems for many diseases such as DR [30], AMD [31], glaucoma [32] and cataract [33]. These diagnostic systems offer the potential to be used in large-scale screening programs, with significant resource savings, as well as freedom from observer bias and fatigue. This section briefly mentions such CAD systems based on ocular imaging. Details are mentioned in Appendix B Details on methods for disease detection. The imaging modalities used by these systems are first introduced below.

### Imaging modalities

**Figure 1:**
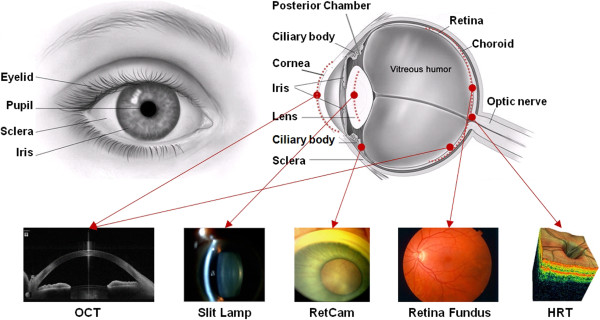
**Ocular Anatomy and various image modalities.** An illustration of the parts of the eye and the imaging modalities associated with them.

Figure 1 shows the anatomy of eye. The visible parts of the eye include the transparent cornea, the sclera, the iris and the pupil. A ray of light, passes through the cornea and anterior chamber, followed by the pupil, the lens and the vitreous before finally focusing on the retina [12].

Various medical imaging devices have been developed to capture the different parts of the eye. These imaging modalities are developed based on various technologies and the captured images are used to observe various pathological signs. Table 1 lists the anatomical structure(s) and the associated disease(s) each imaging modality is able to observe.

**Table 1:** Imaging modalities and diseases to observe

**Imaging modalities**	**Technology**	**Targets**	**Diseases observed**
Retina Fundus	2D; considerably larger areas of the fundus	Interior surface of the eye (retina;	DR, glaucoma, AMD
	than can be seen at one time with handheld	optic disc; macular; posterior pole)	
	ophthalmoscopes		
OCT	3D; high resolution cross-sectional imaging	Cornea thickness, retinal nerve fibre	Glaucoma, macular
		layer tissue, macular thickness	degeneration and edema
Heidelberg Retina	2D; confocal scanning laser ophthalmoscope	Retina	Glaucoma
Tomography (HRT)			
Slit Lamp	2D; high-intensity light source stereoscopic	Eyelid, scelra, conjunctiva, iris,	Cataract
	magnified view of the eye structures	lens, cornea	
RetCam	2D; wide angle imaging	Anterior segment, anterior chamber	Anterior segment lesions,
			Retinopathy of Prematurity
Scanning laser	High resolution cross-sectional imaging	Thickness of RNFL	Glaucoma
polarimetry (SLP)			

Though the eye fundus has been observed since 1850 with the invention of the ophthalmoscope by the German physician Hermann Von Helmholtz [34], it was not until the mid 1920s that the Carl Zeiss Company made available the first commercial fundus camera. In the late 1950s fundus photography became ubiquitous in the practice of ophthalmology for general fundus examination and as a means for recording, storing, and indexing images of a patient with relatively simple and affordable equipment [13]. In recent years, other important imaging modalities, such as fluorescent angiography, stereo fundus photography and confocal laser ophthalmoscopy have appeared to enhance diagnostic and observational capabilities in ophthalmology [35].

**Figure 2:**
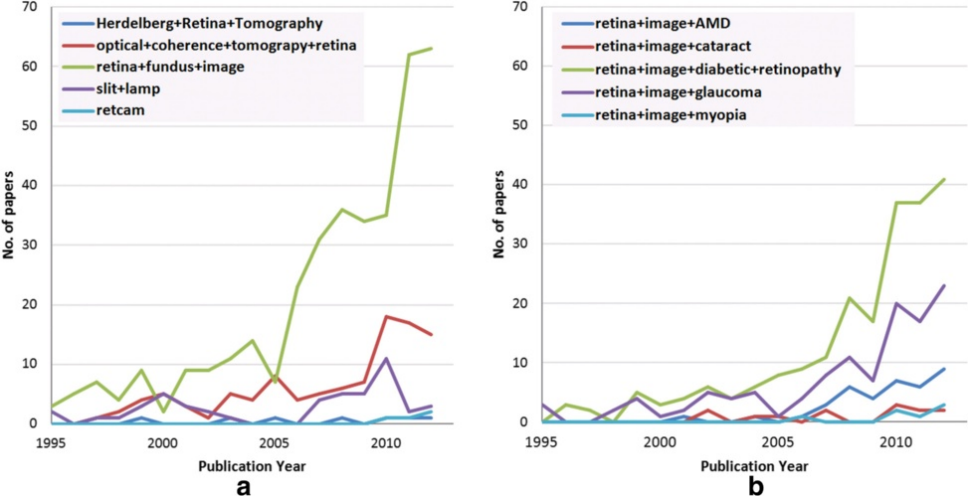
**Publication trends for ocular disease detection. (a)** Number of publications each year for different ocular imaging modality **(b)** Number of publications each year for different ocular disease detection using retinal image (queries to IEEEXplore are as on May 2013).

Major image modalities used for CAD applications and other research trends are shown in Figure 2. These statistics are obtained by searching the *IEEEXplore* publication database and demonstrates the trend of research areas and major imaging modalities for ocular research. Figure 2(a) shows the number of publications related to various ocular imaging modalities, while Figure 2(b) shows the number of publications on CAD for ocular diseases using retinal images. The keywords associated with the search are mentioned in the legend of the corresponding figures. It is observed from Figure 2(a) that of all the imaging modalities, DFP has been attracting the most interest. This observation is further substantiated by a distribution of the works surveyed in this paper (Table 2) wherein the works are arranged according to the disease and the associated imaging modality. Note that imaging modalities or diseases with very few associated works have not been included.

**Table 2:** A distribution of works on CAD of major ocular diseases based on imaging

**Modality**	**AMD**	**Cataract**	**DR**	**Glaucoma**	**PM**
OCT	[31,36]		[37]	[38-40,44-46,49]	[41-43,47,48]
Slit Lamp		[33,50-58]			
SLP				[59-62]	
Retina Fundus	[63-65,74-76,84-86,93-95,102-104,111,112,119-121,127,128,135,136,142-144,147]		[66-68,77-79,87-89,96-98,105-107,113-115,122-124,129-131,137-139,145,146,148-158]	[32,69,70,80-82,90-92,99-101,108-110,116-118,125,126,132-134,140,141]	[20,71-73,83]
HRT				[159-162]	

The possible reasons for this observation are two fold. First, information extracted from the eye fundus could be useful in detecting a variety of diseases such as heart disorders, stroke, hypertension, peripheral vascular disease and DR [13]. Furthermore, the availability of inexpensive fundus imaging cameras makes eye examination simple and cost effective. Another modality which is gaining interest in the research community is OCT. First proposed in 1991 [163], OCT has been widely applied in medical imaging especially for imaging the eye. The most important advantage of OCT compared with DFP is that it provides quantifiable depth information enabling a 3D scan of the target part. Therefore it is possible to detect pathologies with topological changes in-vivo. Although a powerful tool [164], in early years, the progress of OCT-based ocular disease detection has been constrained by the speed of OCT imaging. Early version of OCT required lengthy amounts of time to capture an image. In recent years, with the progress of spectral domain OCT (SD-OCT), which needs only 6 seconds to take a high resolution image, OCT-based ocular disease detection methods are increasing in popularity [165]. A brief description of image databases using DFP and OCT is presented in Appendix A Image databases. In terms of the diseases, the most studied disease is DR, followed by glaucoma and AMD (Figure 2(a)).

The images associated with the above mentioned modalities often need preprocessing to remove noise and improve contrast before they can be analyzed further using CAD methods.

#### Image preprocessing

Some of the common preprocessing methods are histogram equalization [79,87], shade correction [88,89,96], convolution with a Gaussian mask [97], median filtering [98] and blood vessel removal [105,106].

Most of the contrast enhancement techniques use histogram equalization [79,87]. Shade correction is often used to normalize illumination [88,89,96]. For noise reduction, the commonly used techniques are convoluting with a Gaussian mask [97] or using a median filter [98]. Some of the methods also use blood vessel removal as a preprocessing step since they can be detected as false positives while detecting red lesions, especially MAs [105,106].

The choice of a suitable preprocessing method depends on the desired effect. Antal and Hajdu [107] experimentally showed that contrast limited adaptive histogram equalization [113] effectively improves local contrast but also introduces noise. Similarly, vessel removal is used to reduce false positives which can be found during red lesion detection. Considering this subjective nature of the preprocessing methods [107], proposed to choose the best pair of preprocessing and segmentation methods through a fusion algorithm.

The remaining part of this section surveys the works on detecting the major ocular diseases, focusing mainly on DR, PM, AMD and glaucoma since these diseases are investigated more than others. Also, for these diseases, DFP is still the main stream modality, but OCT is rapidly gaining widespread adoption. Therefore we focus on these two modalities. The works on other diseases, such as cataract and corneal opacity, will be reviewed briefly in the section *Other diseases* (Section “Other diseases”).

### Diagnostic methods for diseases

This section briefly introduces causes and symptoms for the major ocular diseases, methods of detecting them from images and a brief discussion on the state-of-the-art and possible future directions. More details on the algorithms are mentioned in Appendix B Details on methods for disease detection.

#### ***Diabetic retinopathy***

##### 

**Figure 3:**
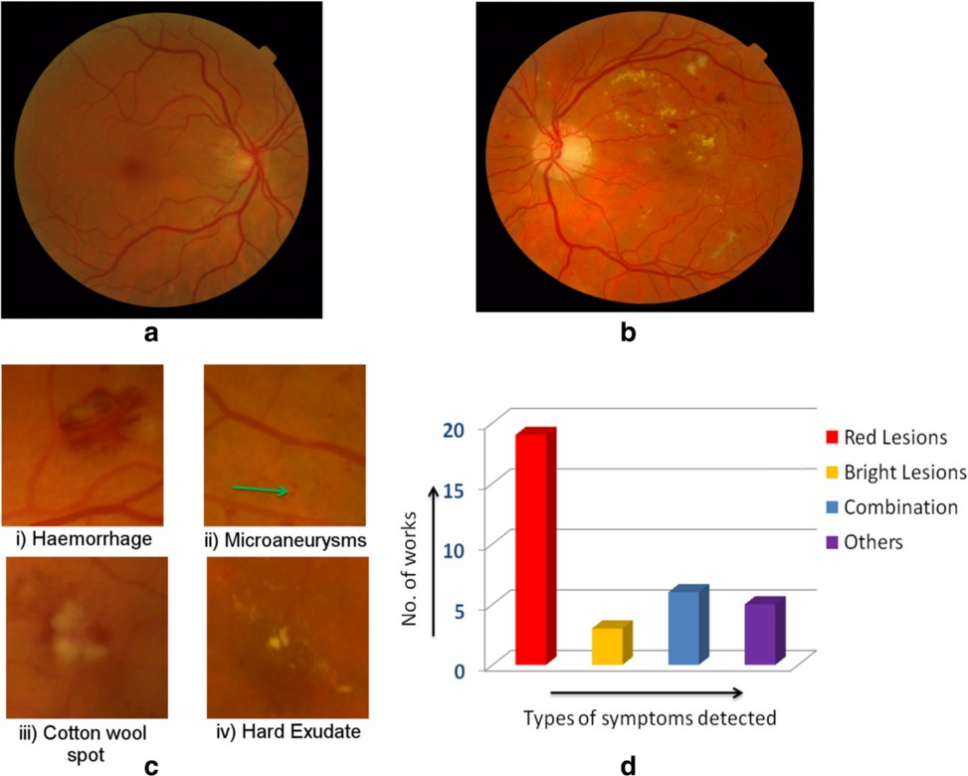
**How does DR look in a DFP. (a)** DFP of a normal eye. **(b)** DFP of an eye affected with DR. **(c)** Common lesions associated with DR. **(d)** A distribution showing number of works detecting each type of symptom.

**Causes and symptoms** DR is a side effect of diabetes which is caused when the blood vessels in the eye start getting blocked due to high sugar content in the blood [166]. Reduced blood supply to the retina can even cause blindness [98]. Symptoms of DR include lesions appearing on the retinal surface. These lesions are visible in a DFP. Figure 3(a) and (b) show the DFPs of a normal eye and a DR affected eye, respectively. DR-related lesions can be categorized into **red lesions** such as Microaneurysms (MA) and Haemorrhages and **bright lesions** such as Hard Exudates (HE) and cotton-wool spots (Figure 3(c)). There are a few works which detect other symptoms as well [146].

##### 

**Detection** Almost all of the work for detecting DR has been performed using DFPs. Most of these approaches detect lesions with special focus on detecting red lesions (Figure 3(d)) especially MAs. MAs receive higher attention since they indicate DR at an early stage [98]. This is important considering that one of the goals for CAD is to provide early detection (Section “Background”). Lesions are detected using morphological operations [114,167] or image filters [130,131]. From our study, we could not find any work on detecting lesions from OCT images.

##### 

**Brief discussion** From the survey of works on DR, it was observed that most of the works have focused on detecting lesions associated with DR. Few works [156] have gone further to convert lesion detection to DR detection. Even for DR detection, most of the works surveyed, have presented their results as a binary detection, i.e whether DR is present or not in an eye. It might be useful to provide a grade to the severity of DR.

In terms of the approach used, only few works [157] have attempted to bypass the lesion detection and used non-clinical features for DR detection. Future research can focus on filling these gaps.

#### ***Glaucoma***

##### 

**Causes and symptoms** Glaucoma is characterized by the progressive degeneration of optic nerve fibres, which leads to structural changes of the optic nerve head, the nerve fibre layer and a simultaneous functional failure of the visual field. As the symptoms only occur when the disease is quite advanced, glaucoma is called the silent thief of sight. Although glaucoma cannot be cured, its progression can be slowed down by treatment. Therefore, timely diagnosis of this disease is important [168,169].

##### 

**Detection** Glaucoma diagnosis is typically based on the medical history, intra-ocular pressure and visual field loss tests together with a manual assessment of the Optic Disc (OD) through ophthalmoscopy. OD or optic nerve head is the location where ganglion cell axons exit the eye to form the optic nerve, through which visual information of the photo-receptors is transmitted to the brain. In 2D images, the OD can be divided into two distinct zones; namely, a central bright zone called the optic cup (in short, cup) and a peripheral region called the neuroretinal rim [90]. Glaucoma causes an enlargement of cup region with respect to OD (thinning of neuroretinal rim) called cupping [69]. This is one of the important indicators and various parameters related to cupping have been used to detect glaucoma.

**Figure 4:**
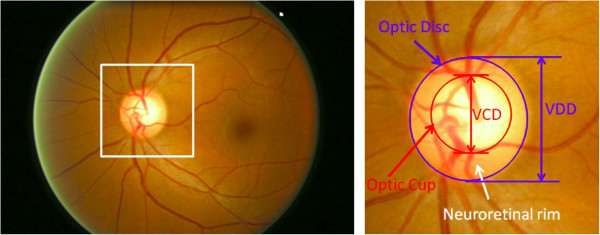
**Major structures of the optic disc in DFP.** The region enclosed by the blue line is the optic disc; the central bright zone enclosed by the red line is the optic cup; and the region between the red and blue lines is the neuroretinal rim.

These parameters include vertical cup to disc ratio (CDR) [170], disc diameter [171,172], ISNT rule [173], peripapillary atrophy (PPA) [174] and notching [175]. The most popular measurement is CDR, which is computed as the ratio of the vertical cup diameter (VCD) to vertical disc diameter (VDD) clinically (Figure 4).

##### 

**Brief discussion** Utilizing DFP and OCT to detect glaucoma are two popular and active directions with OCT having a shorter history. Till now, time-domain OCT and SD-OCT have been widely utilized to perform glaucoma detection [38-40,44-46,49]. However, swept-source OCT (SS-OCT) has not been further exploited for the research of glaucoma. For DFP, the combined analysis of stereo DFP and OCT for extracting disc parameters may boost current performance of state-of-the-art algorithms.

#### ***Age-related macular degeneration (AMD)***

##### 

**Figure 5:**
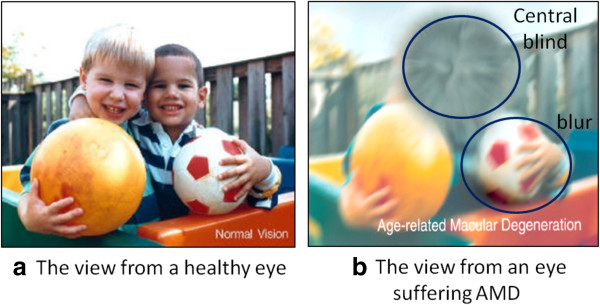
**Vision damage caused by AMD. (a)** Image of a normal eye. **(b)** Image of an eye affected with AMD. (Image taken from Wikipedia http://en.wikipedia.org/wiki/Macular_degeneration).

**Causes and symptoms** AMD causes vision loss at the central region and blur and distortion at the peripheral region (Figure 5). Depending on the presence of exudates, AMD is classified into dry AMD (non-exudative AMD) and wet AMD (exudative AMD). Dry AMD results from atrophy of the retinal pigment epithelial layer below the retina [176]. It causes vision loss through loss of photoreceptors (rods and cones) in the central part of the retina. The major symptom and also the first clinical indicator of dry AMD is drusen, sub-retinal deposits formed by retinal waste. Wet AMD causes vision loss due to abnormal blood vessel growth (choroidal neovascularization) in the choriocapillaris, through Bruch’s membrane, ultimately leading to blood and protein leakage below the macular. Bleeding, leaking, and scarring from these blood vessels eventually cause irreversible damage to the photoreceptors and rapid vision loss if left untreated. The major symptom of wet AMD is exudation [177].

##### 

**Detection** AMD can be detected from DFP, OCT, X-ray, and Magnetic Resonance Imaging (MRI). Among them, DFP is perhaps the most widely used one for AMD detection, while OCT is rapidly growing in use. Most of the approaches detecting AMD from DFPs focus on detecting drusen using local thresholding [63,65], wavelets [63], background modeling [94] and saliency [102] etc. Some of the works have also attempted to bypass drusen detection and directly predict AMD [111,112,119,120,127,128,178]. Considering detecting AMD from OCT, it is easier to observe exudates and edema in OCT images. OCT can segment out retinal layers. Texture and thickness of these layers can help in distinguishing normal region and region corresponding to exudates [31,36].

##### 

**Brief discussion** From the above works, it was observed that although OCT imaging is increasingly prevalent, DFP is still the mainstream image modality for AMD detection and screening. It is an active research avenue. However with the progress of SD-OCT, OCT based AMD detection and screening is emerging as a new area of focus.

#### ***Pathological myopia (PM)***

##### 

**Causes and symptoms** As one of the leading causes of blindness worldwide, Pathological myopia (PM) is a type of severe and progressive nearsightedness characterized by changes in the fundus of the eye, due to posterior staphyloma and deficient corrected acuity. PM is different from myopia which is caused by the lengthening of the eyeball. For myopia both environmental and genetic factors have been associated with its onset and progression [179], while PM is primarily a genetic condition [180]. Unlike myopia, PM is accompanied by degenerative changes in the retina, which if left untreated can lead to irrecoverable vision loss. The accurate detection of PM will enable timely intervention and facilitate better disease management to slow down the progression of the disease.

##### 

**Detection** PM has been detected mostly from DFPs where retinal degeneration is observed in the form of PPA [181,182]. PPA is the thinning of retinal layers around the optic nerve and is characterized by a pigmented ring like structure around the optic disc. Apart from DFPs, there have been studies to detect PM from OCT images [183] however CAD systems for detecting PM from OCT images have not emerged yet.

##### 

**Brief discussion** Ohno-Matsui et al. [47] analyzed the relationship between the shape of the sclera and the myopic retinochoroidal lesions, and concluded that SS-OCT can provide important information on deformations of the sclera which are related to myopic fundus lesions. Such clinical discoveries provide strong evidences for the use of SS-OCT as a good candidate for future PM-CAD development.

#### ***Other diseases***

Other major diseases that may lead to blindness include cataract and corneal opacity. Cataract is characterized by a cloudiness in the lens while corneal opacity finds cloudiness in the cornea. CAD research has been conducted for cataract grading rather than detection using on slit lamp images [33]. Grading of cataract severity is essential for cataract surgical planning [184] and an automated grading system offers an objective and efficient solution. Grading is performed by locating the cloudiness and assessing its opacity level [33]. For corneal opacity, there have not been any automatic detection methods reported so far, to the best of our knowledge.

### Discussion

Feature extraction plays an essential role in ocular image based CAD systems. From the survey, we observe two broad classes of features used in the ocular CAD systems. Approaches using each one of these are described below:

#### ***Approaches using clinical features***

Many of the retinal image based CAD systems employ clinical domain knowledge during the feature selection and decision making processes. Such systems focus on identifying disease associated landmarks from images. A number of clinically relevant features can be extracted from the identified landmarks. For example, the following image cues are highly related to glaucoma: large optic CDR [185]; appearance of optic Disc haemorrhage (DH) [186]; thinning of the neuroretinal rim (NRR) or notching of the NRR [175] and presence of PPA [174]. These features based on clinical knowledge can be described as clinical features.

The early efforts in retinal image analysis were focused on optic disc localization. Lowell et al. [187] used specialized template matching to locate optic disc, followed by a global elliptical and local deformable contour model for disc segmentation. Xu et al. [132] presented a deformable-model-based algorithm for the detection of the optic disc boundary in fundus images. Later efforts were spent in optic cup detection. Abramoff et al. [133] analyzed stereo-based DFPs for rim and cup segmentation via pixel feature classification. Wong et al. [188] detected the optic cup using vessel kinking analysis. Joshi et al. [189] proposed a depth discontinuity (in the retinal surface)-based approach to estimate the cup boundary. Based on cup and disc detection, CDR can be obtained based on which CAD systems for automatic glaucoma detection were developed [32,69,70,80]. Cheng et al. [73,190] developed PPA detection algorithms for Pathological Myopia (PM) detection. Liang et al. [104] focused on detecting drusen presented in retina for automatic AMD detection. Other researchers worked on CAD systems for DR based on various vasculature segmentation algorithms, e.g., matched filters [66,67], vessel tracking [68] or morphological processing [77,78].

The advantages of using clinical features in CAD systems are obvious: the CAD results can be interpreted and presented with clinical meaning, furthermore, the prior knowledge allows modeling the disease detection with a small data set, which is critical when the training data is insufficient.

However, the detection models built using clinical features have a number of limitations as mentioned below: 

• The modeling process is localization or segmentation dependent. For example, [32,69] detect glaucoma based on optic cup and disc segmentation, a small error in disc localization may propagate downstream and finally yield an error in detection.

• The systems are usually threshold-based or rule-based in the decision making stage thus it, by nature, does not produce a quantifiable measurement for the disease detection.

• A model built upon prior knowledge may not evolve with the growing available data.

• As different diseases may possess different landmark features, the system developed for one disease may not be adaptable for other diseases.

• Such systems usually needs to learn from manually curated ground truth images, which is not only time consuming but also prone to human error.

• Finally and most importantly, detection of one particular disease associated landmark may neither be the necessary nor be the sufficient condition for disease detection. For example, [71,83] proposed to recognize PM based on PPA detection, however, having PPA may or may not imply having PM.

Detecting all the retinal changes in DFPs is much more difficult compared to detecting a particular landmark. Statistical learning based on image feature extraction can be a possible solution to address these challenges. The following section casts light on this possibility.

#### ***Approaches using non-clinical features***

With an increasing availability of image databases and advances in statistical learning, new CAD systems are shifting to non-clinical features. Non-clinical image features relate to the *content* of the image such as color, texture and gradient.

Many image feature extraction techniques can be applied to retinal image based CAD systems. Bock et al. [81] used an appearance based approach to quantitatively generate a glaucomatic risk index from retina images. Cheng et al. [91] used Focal Biologically Inspired Feature (FBIF) for glaucoma type classification. Wang et al. [191] presented a DFP mosaic algorithm based on Scale-Invariant Feature Transform (SIFT) feature [192] to overcome low contrast and geometric distortion between different fields of view of DFPs. Extracted SIFT features were described using vectors to determine the matching feature point pairs between two images. The transformation matrix was then computed according to purified matching point to generate a panoramic picture with a wide field of view containing more information which may improve CAD systems. Xu et al. [181] presented a CAD system for PM detection based on SIFT features extracted from a DFP. The system achieved a high AUC value (98.4%) as compared to the earlier approaches to detect PM using particular image cues [83].

Another example is the use of superpixels [193,194]. A superpixel is a perceptually consistent unit with all pixels in a group being similar in color and texture. It reduces the complexity of images from thousands of pixels to only a few hundred superpixels. Algorithms such as Simple Linear Iterative Clustering (SLIC) [195] have been developed to aggregate nearby pixels into superpixels whose boundaries closely match true image boundaries. Many features can be computed from superpixels such as shape, color, location and texture, and they can be used for classification via learning algorithms. Xu et al. [92] presented a superpixel based learning framework based on retinal structure priors for glaucoma detection. The use of superpixels leads to a more descriptive and effective representation than those employed by pixel-based techniques while at the same time yielding significant computational savings over methods based on sliding windows.

Non-clinical features can be considered to be associated with a data driven approach, which has shown many advantages over the approach using clinical features. Extracting non-clinical features is followed by learning from the labeled examples, therefore fewer manual ground truth labeling is needed as compared to the approaches using clinical features. As these systems do not rely on particular image landmarks, they avoid the error cascading due to initial segmentation or localization. Non-clinical features are generalized features which make it possible for the system to transfer knowledge learned from one disease to other diseases. Such feature extraction can facilitate learning algorithms such as multi-task learning [196,197] and transfer learning [198]. Furthermore, since the techniques apply statistical evaluation, the performance of the systems is expected to improve when more data is available. The result of such systems can be a quantifiable score other than Yes or No, which is particularly useful in clinical assessment. The use of non-clinical features for CAD is a promising area for future CAD systems.

## Result: predicting ocular diseases based on genetic information

Genetic information can be used to detect heritable disease related genotypes, mutations or phenotypes for clinical purposes [199]. Ocular diseases are highly inheritable, thus genetic information can provide important insights into disease risk and disease prognosis.

### Heritability of ocular diseases

Heritability is the proportion of phenotypic variation in a population that is attributable to genetic variation among individuals [200].

According to [201], heritability can be presented in statistical terms a linear mixed model, where the observable characteristics of an organism can be represented as a linear function of genetic and environmental factors, namely: *Phenotype*(*P*) = *Genotype*(*G*) + *Environment*(*E*), and the heritability can be represented as *H*^2 ^= *G */*P* where *H*^2^ represents the heritability due to all genetic effects. Since the beginning of the 20th century, heritability studies have been conducted on numerous diverse biological and psychological human traits. Among these, attempts have been made to estimate the genetic contribution to human longevity and lifespan [202,203], and a person’s susceptibility to becoming a smoker [204,205].

In 1992, the first ophthalmic twin study was conducted to investigate the heredity of refractive error [206].

**Figure 6:**
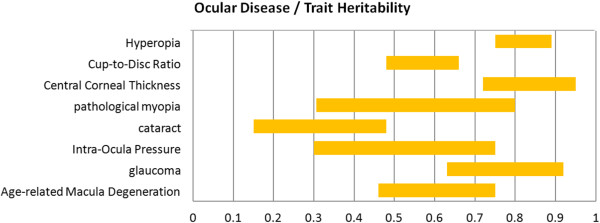
**Heritability for various ocular traits.** The range of heritability values for different ocular traits. A higher heritability value means a higher change of inheriting the trait.

Since then, over 100 articles have been published in the scientific literature examining the genetic contribution to variation in ophthalmic traits. Table 3 summarizes the heritability of various ocular diseases or ocular related phenotypes as reported in the literature. It is observed that the heritability values reported in different studies vary from each other, as the value is population related.The range of heritability values are shown in Figure 6, from which it is observed that Central Corneal Thickness is the most heritable trait while PM spans a wider range due to its population dependence, and cataract seems a less heritable disease.

**Table 3:** Heritability for ocular diseases or disease related traits

**Disease/Traits**	**Heritability value**	**Source**
AMD	0.7	[207]
AMD	0.75	[208]
AMD	0.71	[209]
AMD	0.46-0.71	[210]
AMD	0.45	[211]
AMD (small hard drusen)	0.63	[212]
CCT	0.95	[213]
CCT	0.72	[214]
CDR	0.48	[215]
CDR	0.66	[214]
Corneal astigmatism	0.6	[216]
Corneal curvature	0.71	[216]
Cortical cataract	0.24	[217]
Cortical cataract	0.58	[218]
Glaucoma	0.63	[219]
Glaucoma	0.7	[220]
Glaucoma (shallow anterior chamber)	0.92	[221]
Hyperopia	0.75	[222]
Hyperopia	0.86-0.89	[218]
IOP	0.47-0.51	[223]
IOP	0.3	[224]
IOP	0.36	[215]
IOP	0.56-0.64	[225]
Noncongenital cataract	0.15-0.32	[226]
Nuclear cataract	0.356	[217]
Nuclear cataract	0.48	[227]
Ocular refraction	0.89-0.94	[228]
Pathological Myopia	0.306	[229]
Pathological Myopia	0.8	[230]

### Knowledgebases of genetic markers for ocular diseases

For the past 20 years, biomedical research community has spend huge efforts in identifying genetic markers for heritable diseases, through classical linkage studies [231] or recent Genome-wide association studies [232]. The discovered disease related biomarker include genes, mutations or Single-nucleotide polymorphisms (SNPs). Such valuable knowledge has been continuously accumulated in various biomedical databases which are usually called as *knowledgebases*. This section introduces the knowledgebases highly relevant to this study. 

• OMIM - Online Mendelian Inheritance in Man

• OMIM is a continuously updated catalog of human genes and genetic disorders and traits, with particular focus on the molecular relationship between genetic variation and phenotypic expression [233]. It is thus considered to be a phenotypic companion to the Human Genome Project [234]. As on 8 May 2013, it has more than 14, 000 disease related gene entries in stock.

• GWAS Catalogue - Catalogue of Published Genome-Wide Association Studies (GWAS)

• GWAS is an approach to rapidly scan markers across the complete sets of genome (DNA) of many people to find genetic variations associated with a particular disease [235]. The first GWAS published in 2005 [236] was associated with an ocular disease. It investigated AMD and found two SNPs that are significantly associated with AMD. Since then, similar successes have been reported using GWAS to identify genetic variations that contribute to risk of type 1 diabetes [237], Parkinson’s disease [238], heart disorders [239], obesity [240] etc. The GWAS Catalogue http://www.genome.gov/gwastudies/is a collection of GWAS discovered SNPs, hosted by NHGRI (National Human Genome Research Institute). SNP-trait associations listed in the GWAS Catalogue are limited to those with *p *- *values *< 1.0 × 10^-5^. As on 8 May 2013, the catalog includes 1594 humane GWA studies which examined over 200 diseases and identified more than 10,000 disease associated SNPs.

#### ***Ocular disease related SNPs***

**Figure 7:**
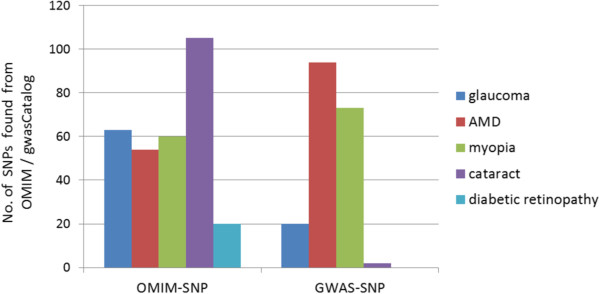
**Ocular disease related SNPs found in OMIM and GWAS Catalogue.** (query made on May 8th, 2013).

Figure 7 shows the ocular disease related SNPs found from the OMIM and GWAS Catalogue knowledgebases. There are potentially many uses of these identified SNPs: a better understanding of disease etiology, personalized medicine, new leads for studying underlying biology and risk prediction. From a risk prediction perspective, it is reasonable to average a larger number of predictors, of which some may have (limited) predictive power, and some actually may be noise. The idea being that when added together, the combined small signals results in a signal that is stronger than the noise from the unrelated predictors [241].

### Discovering novel disease related snps from large-scale genome wide association study

Computational methods investigating for SNP-trait association study [242,243] have been developed. Such methods treat SNPs as individual players in one’s genetic profile. Following these methods, efforts [244-246] have been expanded to investigate those SNPs which have little effects on disease risk individually but influence the disease risk jointly, the phenomenon being known as epistatic interaction, where the effects of one gene are believe to be modified by one or several other genes. The single-locus and epistasis SNP detection based algorithms test individual SNPs or pair of SNPs without taking into consideration, the underlying biological intertwining mechanism. Whereas, the real gene-gene interaction participating in biological pathway are often composed of a group of arbitrary number of SNPs. To date, exhaustively detecting significant SNP groups of arbitrary size is still computationally infeasible [245].

Recently, machine learning especially sparse learning algorithms have been introduced for GWAS data analysis. This is intended to tackle the challenge of identifying a group of *N* potent but interwinely correlated SNPs, some of which may not pass the stringent threshold by themselves. Penalized regression based on Least Absolute Shrinkage Selector Operation (LASSO) [247] have recently been explored for GWAS analysis. Some researchers [248,249] have proposed 2-step approaches for Genome-wide association analysis via shortlisting a group of marginal predictors using penalized likelihood maximization for further higher order interaction detection. Hoggart et al. [250] have proposed a method to simultaneously analyze all SNPs in genome-wide and re-sequencing association studies. D’Angelo [251] have combined LASSO and principal-components analysis for detection of gene-gene interactions in genome-wide association studies. These approaches are not global due to the 2-stage process and none of them have considered incorporating prior knowledge into the model building. Prior knowledge can be combined into GWAS to improve the power of association study [252]. it can also model dependencies and moderate the curse of dimensionality.

## Discussion

From the above survey, two major observations were made. First, there is a trend of transition of the way of acquiring knowledge about CAD from semi-automatic to automatic. The second trend is the integration of heterogeneous data sources. These two trends are discussed in the following subsections.

### The trend of semi-automatic to automatic knowledge acquisition

In the 1970s and 80s, research was focused on constructing knowledge-bases from inputs of physicians [253,254] for CAD tools. Building such systems required a lot human intervention, e.g. experts’ inputs, and can be considered as a ‘semi-automatic’ way for knowledge acquisition. Over the years, the alternative approach of automatic knowledge acquisition without inputs from clinicians or experts, has become more popular [255,256]. One such way of knowledge acquisition is to capture patterns in data using non-clinical features (Section “Approaches using non-clinical features”). This approach offers several advantages: 

• Knowledge-bases derived from datasets are more precise in comparison with knowledge-bases constructed from expert inputs, as the inputs provided by human experts may be vague, due to limited grades of perception [257]. An increased precision of CAD systems will make them more reliable for a mass screening application.

• Knowledge-bases constructed using the automated approach captures empirical evidence in the data. This approach aligns with the trend of evidence-based decision making, which emphasizes on the use of empirical evidence to make clinical decisions [258].

• Medical datasets embed local epidemiological patterns. Hence the derived knowledge-bases can result in more accurate CAD tools, as disease and symptom patterns vary from one region to another [259]. A system learnt using data obtained from a particular region can be expected to be more precise in performing mass screening in that same region. The physician experts on the other hand may not be aware of local trends, especially when they do not have sufficient experience of clinical practice in a particular locality.

### The trend of integration of heterogeneous data sources

One of the reasons, why CAD tools may be found to have sub-optimal accuracy is that the training data may itself lack all the attributes that are required for decision making [260]. Combining decision support methodologies that process information stored in different data formats has been shown to improve the performance [261]. Apart from laboratory information, attributes extracted from gene profiling data, visual clues from medical image, as well as other sources could be combined and may possibly lead to more satisfactory accuracy.

The advances in technologies related to medical signal acquisition, medical imaging and genotyping have resulted in a increased volume and complexity of collected bio & medical data. This makes it difficult for physicians to parse through the information while providing timely diagnoses and prognoses. Due to its complexity, analysis of such data has been limited to bioinformatics applications [262]. There is a significant need for development and improvement of computer-aided detection or decision support systems in medicine, with an expected amplification in the future.

In the era of information explosion, data from multiple sources are becoming increasingly available. Retinal fundus cameras can be found in numerous primary community healthcare institutions as well as optical shops. With the dramatic reduction in genotyping costs in recent years, it is foreseeable that SNP data can be acquired at low cost and with as much as ease as demographic clinical data in the near future. The health screening outreach programs have allowed individuals access the clinical data which was hard-to-access previously.

Each of these heterogeneous data sources (image features, personal profile data, SNP data) is likely to contain a different perspective on the disease risk of an individual, based on the pathological, environmental and genetic mechanisms of the disease. These perspectives may potentially be complementary and a combination of the data from these independent sources can provide a more comprehensive and holistic assessment of the disease.

Integration of different data sources in CAD systems can also help in early detection since some of the early symptoms of the disease may appear in one data source but not the other. Consequently, using just one single source or type of data may be limiting for early detection.

There is no previous work attempting to combine these three types of data for automatic disease detection except [20] mentioned in Section “Result: CAD of ocular diseases based on clinical data”. Possible reasons could be that only until recently such data has become available on a large scale. Also, researchers working on these heterogeneous data sets usually come from different domains with different foci, e.g. computer vision and image understanding researchers focused on DFP analysis, bioinformaticians are interested in discovering disease associated SNP or SNP groups. Effectively combining these data can maximize the information gain and pave the way for a holistic approach for automatic and objective disease detection and screening.

Converse to the integration of multiple data sources, there is a possibility of using the same image to detect multiple diseases since many ocular diseases may have common symptoms. Along this line, there are already machine learning algorithm such as multi-task learning which look to solve similar problems. However, to the best of our knowledge, currently there is no work in this direction.

## Conclusion

CAD for ocular diseases, which can automate the detection process, has attracted extensive attention from many clinicians and researchers. They not only alleviate the increasing burden on the clinicians by providing automatic and objective diagnosis with valuable insights, but also offer early detection and easy access for patients. In this article, we have reviewed in detail the recent progress of developed methods used in CAD of ocular diseases in available literature. We investigated three types of data (i.e., clinical, genetic and imaging) that have been commonly used in existing methods for CAD. A number of major ocular diseass including DR, Glaucoma, AMD and PM were also introduced along with existing methods that have been proposed to detect these diseases. The necessity of turning semi-automatic acquisition of domain knowledge into fully automatic ones (which does not require inputs from operators) was examined. The advantages of integrating heterogeneous data sources for ocular disease detection were highlighted. We are of the belief that these two trends are of great importance and deserve further study in the future.

## Appendix

### A Image databases

This section briefly describes the commonly used databases for each disease. The name of the associated disease is mentioned in brackets after the name of the database. 

• *ORIGA*^-*light*^ (Glaucoma): The *ORIGA*^-*light*^[263] database contains 650 annotated DFPs, including 168 glaucomatous images and 482 randomly selected nonglaucoma images. Each image is tagged with grading information, and manually segmented result of optic disc and cup.

• Erlangen Glaucoma Registry (Glaucoma): The Erlangen Glaucoma Registry [264] includes 861 eyes of 454 Caucasian subjects (239 normal eyes of 121 subjects, 250 ocular hypertensive eyes of 118 patients, 372 eyes of 215 patients with chronic open-angle glaucoma).

• The Singapore Malay eye study (SiMES) (Glaucoma): SiMES [24] is a population-based study conducted from 2004 to 2007 to assess the causes and risk factors of blindness and visual impairment in the Singapore Malay community. The study was approved by the institutional review board of Singapore Eye Research Institute. The database contains 3280 subjects, with complete or partial personal data, DFP data and genome information for each subject. The personal data in SiMES contains demographic data such as age, gender and height, ocular examination data, such as IOP and cornea thickness, as well as historical medical data. SiMES examined a population-based, cross-sectional, age stratified, random sample of 3280 Malays (78.7% participation rate) aged 40 to 80 years living in Singapore.

• The Singapore Indian Eye Study (SINDI) (Glaucoma): The SINDI [25] is a population-based, cross-sectional study, which was conducted on 3400 Indians aged 40 to 83 years residing in Singapore. Ocular components including axial length (AL), anterior chamber depth (ACD), and corneal radius (CR) were measured by partial coherence interferometry. Refraction was recorded in spherical equivalent (SE). After 502 individuals with previous cataract surgery were excluded, ocular biometric data on 2785 adults were analyzed.

• The Singapore Chinese Eye Study (SCES) (Glaucoma): The aims of SCES [26] are to identify the determinants of Anterior Chamber Depth (ACD) and to ascertain the relative importance of these determinants in Chinese persons in Singapore. 1060 Chinese participants were recruited from the Singapore Chinese Eye Study. All subjects underwent AS optical coherence tomography (OCT; Carl Zeiss Meditec, Dublin, CA). Customized software (Zhongshan Angle Assessment Program, Guangzhou, China) was used to measure the AS-OCT parameters. Anterior chamber depth was determined using IOLMaster (Carl Zeiss Meditec). Univariate and multivariate regression analysis were performed to assess the association between ACD with ocular biometric and systemic parameters.

• High-Resolution Fundus (HRF) Image Database (Glaucoma): The HRF [265] database has been established by Friedrich-Alexander University Erlangen-Nuremberg (Germany) and the Brno University of Technology (Czech Republic). contains 15 images of healthy patients, 15 images of patients with DR and 15 images of glaucomatous patients. Binary gold standard vessel segmentation images are available for each image. Masks determining field of view (FOV) are provided for particular datasets. The gold standard data is generated by a group of experts working in the field of retinal image analysis and clinicians from the cooperating ophthalmology clinics.

• The Rotterdam Study (Glaucoma): The Rotterdam Study [266] is a prospective population-based cohort study investigating age-related disorders. The study started in 1990 and is still ongoing. The original cohort was comprised of 7983 participants 55 years or older; ancillary studies were added later on, and in total 14,926 participants have been enrolled. In 2007, OCT scanning of the macular and ONH regions was added to the armamentarium. To determine which regions of the OCT volumes could be segmented in what fraction of subjects, the macular and ONH of 925 consecutive subjects was imaged with the Topcon 3-D OCT-1000 (Topcon, Tokyo, Japan).

• DIARETDB0 and DIARETDB1 (DR): These two databases [267,268] of DFPs contain wide variety of DR related lesions such as Hemorrhages (H), Microaneurysms, Hard Exudates (HE), Cotton Wool Spots (CWS) or Soft Exudates and Neovascularization. There are 219 images in total with 25 of them completely normal. The Field of View (FOV) is 50 deg and image resolution is 1500×1152 pixels. The ground truth is in the form of locations and sizes of the lesions. The major difference between the two databases is that DIARETDB0 has *calibration level 0* DFPs which means that the images are taken with different fundus cameras with unknown camera settings. However DIARETDB1 has *calibration level 1* DFPs in a sense that images are taken from the same fundus camera. DIARETDB0 is supposed to have more variation in visual appearance across images as compared to DIARETDB1.

• ROC (DR): ROC stands for Retinopathy Online Challenge [269] which is a competition aiming to compare the accuracies of MA detectors on a benchmark database. The database consists of 50 training and 50 testing images. The ground truth consists of the positions of the centers of MAs and irrelevant lesions. Ground truth for the training images is released while that for the test images is kept with the organizers. Participants can submit their detection results through the challenge website and the organizers compute a performance score for the detections.

• Messidor (DR): Messidor database [270] consists of 1200 DFPs containing MAs, Neovascularization and Hemorrhages. The images were acquired using a color video 3CCD camera on a Topcon TRC NW6 non-mydriatic retinograph with a 45 degree FOV. The images are of resolution 1440 × 960, 2240 × 1488 or 2304×1536 pixels. The ground truth is in the form of Retinopathy grade from 0 (normal) to 3 (most severe). Similarly, risk of macular edema is marked on a scale from 0 (no risk) to 2 (high risk).

• STARE (DR, AMD): (STructured Analysis of the REtina) is a dataset containing images of multiple diseases. It contains 397 DFPs in total and ground truth is in the form of severity grades for the disease. The images are of resolution 700×605. Of all the images, 62 were labeled as containing drusen, including 20 ones as large many, 13 ones as large few, 10 as fine many, and 19 as fine few. To the best of our knowledge, it is the first dataset containing drusen labeling. STARE also contains DR related lesions. 91 images are labeled as being affected by DR [75]. It also contains manually labeling of vessels of part of the images.

• ARIA (DR, AMD): ARIA was published by St Paul’s Eye Unit of Royal Liverpool University Hospital Trust in UK. It contains 212 images in total, including 92 ones with AMD, 61 normal ones, and 59 ones with DR.

• AREDS (AMD): Age-Related Eye Disease Study (AREDS) enrolled 4,757 participants, aged 55-80 years. Among them, 3640 participants had at least early AMD and the other 1117 ones did not [271].

• Thalia-D (AMD): Thalia is a dataset constructed by iMED group from *I*^2^*R* (Institute of Infocomm Research, Singapore). It consists of 350 images, with 96 labeled as early AMD (drusen) and the others non-AMD (no drusen). Image resolution is 3072×2048 and ground truth is in the form of marked drusen boundary [272].

• EUGENDA (AMD): Euregio genetic database (EUGENDA) is an ongoing project currently targets on AMD. Now it contains more than 4000 images with more than 191 ones containing drusen (http://www.eugenda.org/).

• CAPT (AMD): Complications of Age-Related Macular Degeneration Prevention Trial (CAPT) is a randomized clinical trial to evaluate whether prophylactic laser treatment to the retina can prevent the complications of the advanced stage of AMD. In total, 1052 patients with two high-risk eyes were enrolled. The images collected by CAPT can be used as dataset for automatic AMD detection [273].

Note that for Pathological Myopia, to the best of our knowledge, there have not been many studies on image based CAD. However, there were studies on the prevalence rate of PM [274-277] which used large volumes of DFPs.

### B Details on methods for disease detection

#### Diabetic retinopathy

##### ***DFP for Detecting DR***

Detection of DR using DFP typically involves four steps 1) Preprocessing to enhance lesions, 2) Segmentation of candidate lesions, 3) Feature extraction from candidate lesions 4) Classification of candidate lesions into lesions and non-lesions, based on the features extracted. The green channel of the DFP is preferred for analysis since the retina has a good contrast in this channel [98]. Out of these, the segmentation methods specific to DR are discussed below.

Segmentation is usually based on morphological operations [114,167]. Lay and Baudoin et al. [114] were among the first to propose automatic segmentation of MAs. They performed morphological opening of images using structuring elements of different orientations and subtracted the resultant image from the original one, though it is hard to choose an optimal size of the structuring element [97].

Apart from morphological approaches, researchers have used Gabor filters [130], Gaussian correlation filters [131], curvelet transforms [105], wavelet transforms [278], local image properties [279,280], or just the intensity values in the green channel [97,137] for segmenting out candidate lesions.

Some of the works detected both bright and red lesions [106,137,149,153,154] while Abramoff et al. [155] and Agurto et al. [146] have also detected neovascularization in addition to the lesions. Individual detections were then fused in these works to predict the severity of DR.

In terms of the effectiveness of CAD systems for mass screening of DR, it can be assessed by the accuracy of these systems. Accuracy of systems depend on the kind of data used for training and testing them. The Retinopathy Online Challenge (ROC) is aimed at evaluating the accuracy of MA detectors on a benchmark database. The final score of a method is computed by averaging the sensitivities at seven false positive rates (1/8, 1/4, 1/2, 1, 2, 4, and 8 false positives per image). The state of the art score on the ROC database is 0.434 achieved by [79].

##### ***OCT Imaging for detecting DR***

Apart from DFPs, OCT images can also be used for DR detection. An OCT image can analyze different layers of the retina and has the capability of detecting cystoid fluids. Wilkins et al. [37] proposed to detect Cystoid Macular Edema (CME) which is one of the symptoms of DR. They presented a method for segmenting retinal cyst without going further for DR detection. A drawback with the OCT images is that they are prone to noise during capture and a poor Signal to Noise Ratio (SNR) can affect the segmentation accuracy [37].

#### Glaucoma

For glaucoma assessment, there exist mainly four imaging modalities which provide quantitative parameters of the ONH in glaucoma: 1/ Digital Fundus Photograph (DFP); 2/ OCT; 3/ Confocal Scanning Laser Ophthalmoscopy (CSLO) and 4/ Scanning Laser Polarimetry (SLP).

##### ***DFP for detecting glaucoma***

Digital Fundus Photograph (DFP) is one of the main and popular modalities to diagnose glaucoma. Since it is possible to acquire DFPs in a noninvasive manner which is suitable for large scale screening, DFP has emerged as a preferred modality for large-scale glaucoma screening. In a glaucoma screening program, an automated system decides whether or not any signs of suspicious for glaucoma are present in an image. Only those images deemed suspect by the system will be passed to ophthalmologists for further examination.

Glaucoma detection based on DFP can be categorized into three main strategies: 1) detection without disc parametrization, 2) detection with disc parametrization using stereo DFP, and 3) detection with disc parametrization with monocular DFP.

For detecting glaucoma without disc parametrization, a set of features are computed at the image-level without performing OD and cup segmentation from the DFP. Then, two-class classification is employed to classify a given image as normal or glaucomatous. Bock et al. [90] presented an automated glaucoma detection system, where different generic feature types were compressed by an appearance-based dimension reduction technique. A probabilistic two-stage classification scheme combined these features types to extract the novel Glaucoma Risk Index(GRI). Several other papers [81,82,99-101,108] have also adopted this strategy for glaucoma detection.

For the other two strategies of detecting glaucoma with disc parametrization, OD and cup regions are segmented to estimate the relevant disc parameters. The strategy based on monocular DFP utilizes the 2-D projection of retinal structures to compute the areas of OD and cup. As shown in Figure 4, in a monocular DFP, OD appears as a bright circular or elliptic region partially occluded by blood vessels. Retinal nerve fibres converge to the OD and form a cup-shaped region known as the cup. After segmenting the OD and cup [92,109,116], vertical CDR is estimated to detect glaucoma [80,117,118,125,126]. In a recent work [117], Cheng et al. introduced optic disc and optic cup segmentation using superpixel classification for glaucoma screening. In optic disc segmentation, histograms and centre surround statistics were used to classify each superpixel as disc or non-disc. For optic cup segmentation, in addition to the histograms and centre surround statistics, the location information was also included into the feature space to boost the performance.

Different from monocular DFP, a stereo set of DFP contains partial depth information, which can be used to characterize the region inside the OD such as the cup and neuroretinal rim. A considerable body of work based on stereo DFP has been carried out to detect glaucoma [110,132-134,140,141]. For example, Abramoff et al. [133] proposed an automated segmentation method of the optic disc cup and rim from stereo color photographs using pixel feature classification. In their system, a depth map and outputs of a Gaussian steerable filter bank were used as features for training a classifier.

##### ***OCT Imaging for detecting glaucoma***

**Figure 8:**
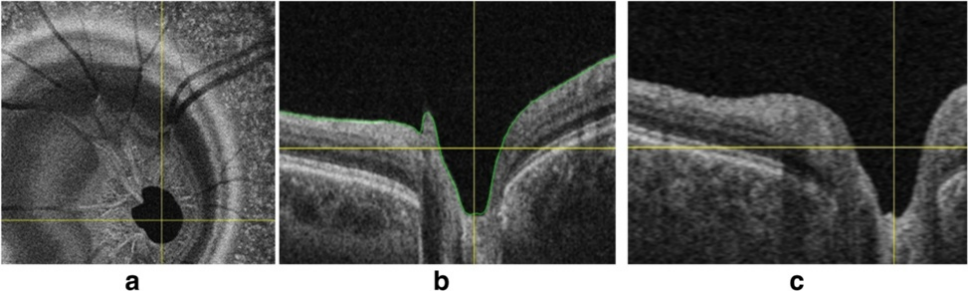
**Cross-sectional images of the spectral-domain OCT volume in glaucoma. (a)** X-Y image of the OCT volume. **(b)** X-Z image of the OCT volume corresponding to the horizontal line in **(a)**. **(c)** Y-Z image of the OCT volume corresponding to the vertical line in **(a)**.

OCT is relatively new in ophthalmic care compared to fundus photography. And the use of image analysis techniques based on OCT images has a shorter history. Nevertheless, it is a rapidly growing and important modality for glaucoma detection. In the assessment of glaucoma, the optic disc is an important structure. While stereo fundus photography is able to extract some 3-D shape information of the optic nerve head, OCT provides true 3-D information. Figure 8 gives three spectral-domain OCT images in glaucoma [44]. There are mainly two strategies for segmenting the disc/cup in optic-nerve head (ONH) from OCT images for glaucoma detection [12]: 1) a pixel classification approach applied to depth-columns of OCT voxels in which the reference standard is defined by manual planimetry from stereo fundus photographs and 2) direct segmentation of structures (neural canal opening and cup) from 3-D OCT images using a graph theoretic approach.

For the first strategy of segmenting ONH, a series of studies [44-46] has been performed. Lee et al. [45] developed a method which can segment the optic disc cup and neuroretinal rim in spectral-domain OCT scans centered on the optic nerve head. Their system first segmented 3 intraretinal surfaces using a fast multiscale 3-D graph search method. Then, the retina of the OCT volume was flattened to have a consistent shape across scans and patients based on one of the segmented surfaces. Finally, selected features derived from OCT voxel intensities and intraretinal surfaces were used to train a k-NN classifier, which determined which A-scans in the OCT volume belong to the background, optic disc cup and neuroretinal rim. As a further study, [44] presented a fast, fully automatic method to segment the optic disc cup and rim in 3-D SD-OCT volumes, in which automated planimetry was performed directly from close-to-isotropic SD-OCT scans. In their proposed scheme, four intraretinal surfaces were segmented by utilizing a fast multiscale 3-D graph search algorithm. Then, the retina in each 3-D OCT scan was flattened to ensure a consistent optic nerve head shape. For the classifier training, a set of 15 features derived from the segmented intraretinal surfaces and voxel intensities in the SD-OCT volume were selected. Finally, based on the convex hull-based method, prior knowledge about the shapes of the cup and rim was incorporated into the system.

For the second strategy of segmenting ONH, a variety of studies [38-40,49] directly segmented the neural canal opening and cup from 3-D OCT images. Hu et al. [38] introduced a scheme for segmenting the optic disc margin of ONH in SD-OCT images using a graph-theoretic approach. They utilized a small number of slices surrounding the Bruch’s Membrane Opening (BMO) plane for creating planar 2-D projection images. In addition, since there are large vessels in images, the information from the segmented vessels was used to suppress the vasculature influence by modifying the polar cost function and remedy the segmentation difficulty. In order to investigate the correspondence and discrepancy between the Neural Canal Opening (NCO)-based metrics and the clinical disc margin, Hu et al. [40] proposed an automated approach for segmenting the NCO and cup at the level of the Retinal Pigment Epithelium (RPE)/Bruch’s Membrane (BM) complex in SD-OCT volumes.

##### ***CSLO Imaging for detecting glaucoma***

**Figure 9:**
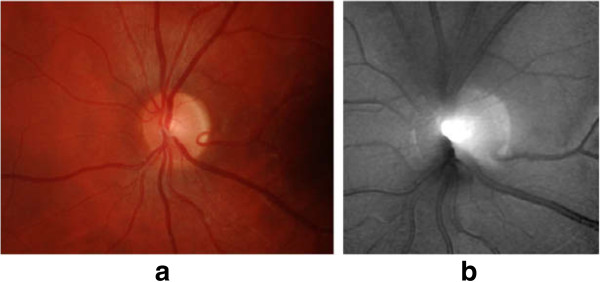
**Example images of the central retina.** Optic nerve head (ONH) centred fundus photograph **(a)** is used for automated glaucoma detection by the proposed glaucoma risk index while glaucoma probability score utilizes HRT 2.5-dimensional topography images **(b)** Images taken from [90].

CSLO utilizes a diode-laser light source to produce quantitative measurements of the ONH and posterior segment. A commercially available CSLO device is the Heidelberg Retina Tomograph (HRT; Heidelberg Engineering, Heidelberg, Germany), which is capable of detecting the structural alterations in glaucoma. An example of an HRT image is shown in Figure 9(b) [90].

Numerous studies [159-162] have reported that HRT measurements are highly reproducible. In [161,162], after outlining the optic disc border manually, the system generated geometric parameters such as the cup volume, cup depth, cup shape measure or even retinal height variations along the rim contour. Then, they applied discriminant analysis (Moorfields Regression Analysis (MRA)) to combine these geometric parameters. Since the gained quantitative parameters are not fully objective due to the manual outlining of the OD border, Burgansky-Eliash et al. [159] used the parameters of a non-linear shape model of the topographic ONH shape for glaucoma classification, which overcame the subjectivity of contour based methods. In the work of [160], the progression of glaucomatous degeneration over years could be quantified. The authors utilized the HRT Topographic Change Analysis (TCA) to automatically locate and quantify the temporal glaucomatous structural ONH changes.

##### ***SLP Imaging for detecting glaucoma***

SLP is another available imaging modality for the detection of glaucoma. Alongside the structural changing of the ONH, the degeneration of the nerve fibres is depicted by a thinning of the retinal nerve fibre layer (RNFL) in the course of glaucoma. SLP is able to measure the thickness of the RNFL for glaucoma detection. In SLP, the retina is illuminated by polarized light and RNFL thickness can be directly determined from the polarization change of the reflected light [59].

**Figure 10:**
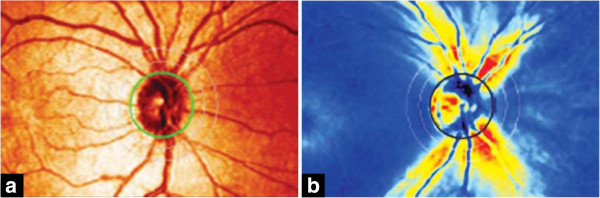
**Images generated by the GDx VCC. (a)** The reflectance image, which is displayed as a colored intensity map (greater reflectance corresponds to a lighter color). **(b)** The retardation map converted to RNFL thickness. The RNFL thickness is color-coded based on the color spectrum with thinner regions displayed in blue and green and thicker regions displayed in yellow and red [281].

SLP is commercialized as the GDxVCC (Carl Zeiss Meditec, Inc., Dublin, CA). GDxVCC includes both the scanner itself and a software program that assists in the acquisition procedure, which can be used to analyze the scan, derives various parameters and translates these into an overall score, the Nerve Fiber Indicator. It could be considered as a soft classification of glaucoma likelihood. Images generated by the GDx VCC are shown in Figure 10[281]. Many glaucomatous progression detection strategies can be formulated for SLP data. Based on repeated GDxVCC SLP measurements, Vermeer et al. [61] tested several strategies to identify the optimal one for clinical use. Medeiros et al. [62] presented a scheme for differentiating between glaucomatous and control cases, which extracted global and sectoral geometric parameters such as average thickness, minimum thickness from RNFL thickness.

#### Age-related macular degeneration

##### ***DFP for detecting AMD***

The existing automatic AMD detection methods focus mainly on detecting drusen, the symptom of early AMD. Several other methods walk a step further to grade AMD.

**Figure 11:**
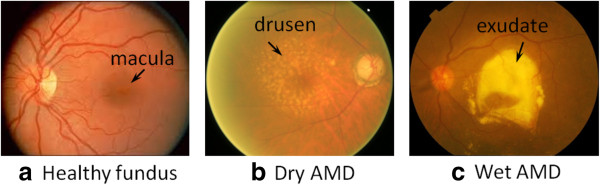
**The symptoms of AMD seen in DFP. (a)** DFP of a healthy eye. **(b)** DFP of an eye affected with dry AMD, with drusen presented. **(c)** DFP of an eye affected with wet AMD. Presence of exudates can be seen.

In DFPs, drusen appear as small bright spot with particular size and orientation, as shown in Figure 11(b). Because the intensity and color of the image may vary with different imaging condition, finding local maxima is a more effective method than global thresholding is. Local maxima are found through geodesic method [63], Histogram based Adaptive Local Thresholding (HALT) [65], and Otsu method based adaptive threshold [74]. After maxima detection, the candidates are further classified according to contrast, size and shape.

Apart from spatial domain, frequency domain has also been used for drusen detection. For example, multi-scale and multi-orientation wavelet is used to detect drusen in a hierarchical framework [63] or through Support Vector Data Description (SVDD), which is derived from support vector machine [76]. Furthermore, a mathematical technique, amplitude-modulation frequency modulation (AM-FM) was shown to be able to generate multi-scale features for classifying pathological structures, such as drusen, on a retinal image [84].

In recent years, with the progress of computer vision and machine learning, more and more advanced techniques have been introduced for drusen detection, e.g., novel feature descriptor such as ICA [85] and biologically inspired features [76], feature selection schemes such as AdaBoost [86], and parameter choosing approaches [64]. A latest work, Thalia [272] is a system for drusen lesion image detection and AMD assessment, using a hierarchical word transform (HWI) as representation.

There are other methods using background modeling [94] and saliency [102]. The background modeling method [94] first segments the healthy structure of eye and blood vessels and the inverse of the healthy parts provide the drusen detection result. The saliency based method [102] first detects the salient regions and then classifies them as blood vessel, hard exudates or drusen. In [95], a general framework was proposed to detect and characterize target lesions concurrently. In the framework, a feature space, including the confounders of both true positive (e.g., drusen near to other drusen) and false positive samples (e.g., blood vessels), is automatically derived from a set of reference image samples. Subsequently a Haar filter was used to build the transformation space and Principal Component Analysis (PCA) was used to generate the optimal filter.

Since drusen is one of the main early symptom of AMD, most of the existing work on AMD detection take drusen detection and segmentation as basis. The overlap of drusen with macular is used to measure the severity of AMD [103,104]. The performance of such methods is restricted by the accuracy of drusen detection. To bypass drusen detection and segmentation, in recent years, researchers have started to seek for methods detecting AMD directly from DFPs. An early attempt in this direction was a histogram based representation followed by Case -Based Reasoning [111]. Good results were produced, however observations indicated that relying on the retinal image colour distribution alone was not sufficient. Thus the authors upgraded the method by using a spatial histogram technique that included colour and spatial information [112]. The latest work from the same team comprises hierarchical image decomposition stored in a tree structure to which a weighted frequent sub-tree mining algorithm is applied. The identified sub-graphs are then incorporated into a feature vector representation (one vector per image) to which classification techniques can be applied [119,120]. These methods detect AMD from the scope of a single image. Another strategy is to use content-based image retrieval. Region based and lesion based features were tested and gave satisfactory performance [127] and [128].

The above mentioned works detect dry (non-exudate) AMD. Till now, there are few works on wet AMD detection except the one proposed in [121] where the basic idea is that the vessels in the DFP seem different under dry and wet AMD. Thus the method first detected the vessels, using a wavelet based method. Subsequently the area, standard deviation, and other features describing the distribution of the vessels were used as features for classification.

##### ***OCT imaging for detecting AMD***

**Figure 12:**
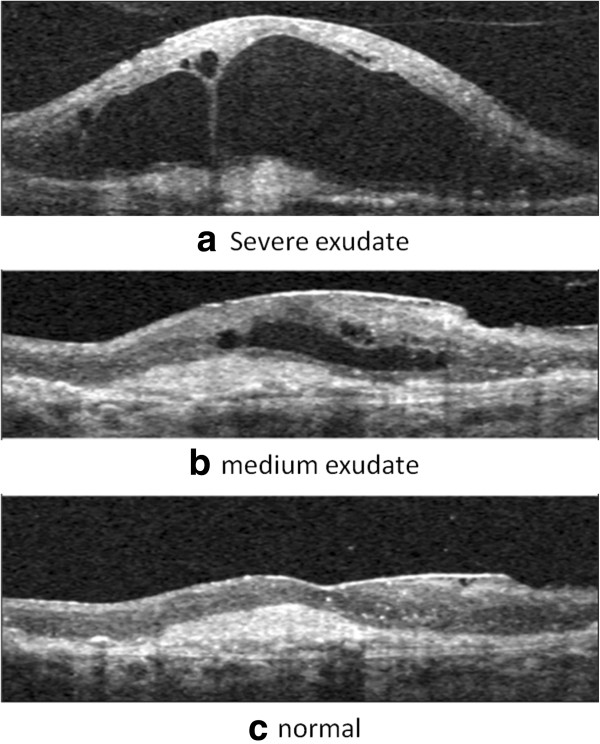
**Example of AMD related exudate in OCT image. (a)** OCT image showing an eye with severe exudate. **(b)** OCT image showing an eye with medium exudate. **(c)** OCT image showing a normal eye.

As mentioned in Section Imaging modalities, it is easier to observe edema and exudates in OCT (Figure 12). In [31], a method for automated characterization of the normal macular appearance in SD-OCT volumes was reported together with a general approach for local retinal abnormality detection. Ten intraretinal layers were automatically segmented and the 3-D image dataset was flattened to remove motion-based artifacts. From the flattened OCT data, 23 features were extracted in each layer locally to characterize texture and thickness properties across the macular. The normal ranges of layer-specific feature variations have been derived from 13 SD-OCT volumes depicting normal retinas. Abnormalities were then detected by classifying the local differences between the normal appearance and the retinal measures in question. This approach was applied to determine footprints of fluid-filled regions-SEADs (Symptomatic Exudate-Associated Derangements) in 78 SD-OCT volumes from 23 repeatedly imaged patients with choroidal neovascularization (CNV), intra, and sub-retinal fluid and pigment epithelial detachment. In [36], the authors improved this method by employing a probabilistically constrained combined graph search-graph cut method refines the candidate SEADs by integrating the candidate volumes into the graph cut cost function as probability constraints.

#### Pathological myopia

Research on CAD of PM has mainly relied on DFP but recently there have been efforts to explore the use of SS-OCT for PM analysis.

##### ***DFP for detecting PM***

An observable sign for PM detection is PPA, an atrophy of pre-existing retina tissue. The APAMEA system proposed by Liu et al. [71] was the first CAD system for PM detection. In APAMEA, features were extracted from a sectional texture map generated from entropy analysis in the optic disc ROI, and SVM learning achieved a 85% specificity and 90% sensitivity. Later on, Tan et al. [72] reported a PPA detection method using a variational level set approach. The method used a disc difference approach to locate PPA by obtain a difference in the two areas, e.g., optic disc with PPA and the fundamental optic disc. It reported a 95% accuracy. The above two methods were based on a rather small data set of only 40 images. A recent advance in PPA detection was reported in [73], which was tested on a much larger dataset containing 1584 images. The authors presented a biologically inspired feature (BIF) approach for the detection of PPA. BIF mimics the cortical processes for visual perception. In the approach, a focal region (ROI) is segmented from the retinal image and the BIF is extracted followed by selective pair-wise discriminant analysis for negative and positive sparse transfer learning. The authors reported that negative sparse transfer learning is superior to the positive one for their task. The method achieves an accuracy of more than 90% in detecting PPA.

Different features have been extracted from DFP for PM detection. APAMEA extracted a texture feature obtained through entropy analysis. In [73] BIF was used for sparse learning. The study conducted by Zhang et al. [20] developed a combined approach integrating SIFT features extracted from DFP with genetic information as well as other clinical data. The study demonstrated that, by learning from multiple data sources, the classifier can achieve a more accurate prediction result. It is the first reported study to combine heterogeneous data including image, genetic and text data for PM detection.

##### ***SS-OCT imaging for detecting PM***

SS-OCT uses a frequency swept laser as a light source [41] and, in practice, has less roll-off of sensitivity with tissue depth than conventional SD-OCT instruments. The current SS-OCT instruments use a longer wavelength, generally in the 1 *μm* range, which has improved their ability to penetrate deeper into tissues than the conventional SD-OCT instruments [282]. Though CAD systems based on SS-OCT have not emerged, some clinical studies have discovered that SS-OCT could be a powerful machine for PM analysis. A recent study conducted in Japan [48] reported that SS-OCT can detect optic nerve pits or pit-like changes in PM eyes. Such changes are not detectable by other imaging modalities.

#### Other diseases

A brief review of cataract grading and CAD for corneal opacity is given below.

##### ***Cataract***

Cataract is characterized by a cloudiness (opacity) in the eye lens which obstructs vision and can even lead to blindness. Cataract can be categorized into three types based on the location of opacity within the lens structure: nuclear, cortical and Posterior Sub-Capsular (PSC) [283]. Nuclear cataract (NC) begins at the center of the lens and spreads towards the surface. Cortical cataract begins at the outer rim of the lens and moves towards the center. PSC forms at the back of the lens. NC is graded using slit-lamp images of the eye while Cortical cataract (CC) and PSC are graded from the retro-illumination images of the eye lens. The grades are usually real numbers in a range that depends on the grading system used.

**Figure 13:**
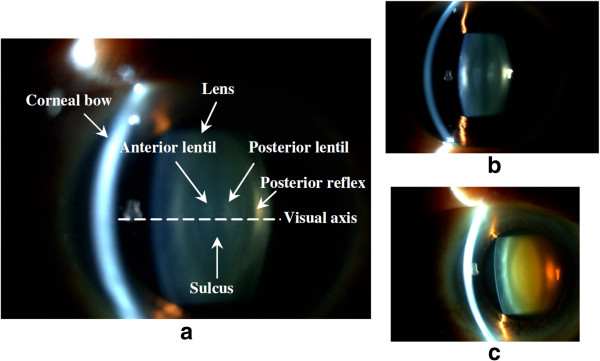
**Parts of the right eye as viewed in a slit-lamp image **[50]**. (a)** Important parts of the eye as seen in a slit lamp image. **(b)** Slit lamp image of a normal eye. **(c)** Slit lamp image of an eye affected with Nuclear Cataract.

**Figure 14:**
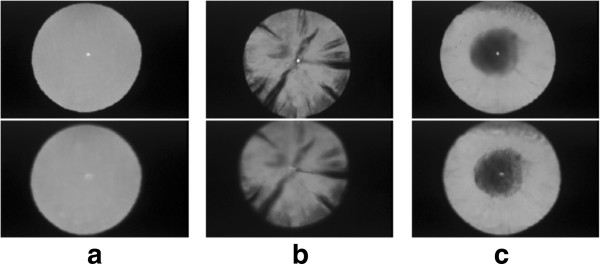
**Examples of retroillumination images.** Retroillumination images corresponding to **(a)** a normal eye lens, **(b)** lens with 61.07% of cortical cataract, and **(c)** lens with 4.95% of cortical opacities and 31.28% of PSC opacities. Top row shows anterior images while the bottom row shows posterior images [58].

Figure 13(b) and (c) show the slit-lamp images of a normal eye and NC affected eye, respectively. It can be seen that the lens nucleus is the affected part and consequently NC is graded by extracting features from the eye lens. The extracted features include intensity of the sulcus region [52] (Figure 13(a)), luminance profile in the eye lens [51] and color and intensity based features extracted from the nuclear region [50,284]. The accuracy of NC grading can be quantified using the average grading difference which is the average of the difference between actual and predicted grading over all the test samples. A lower value of this measure is better. The average grading difference of the state-of-the-art work [284] is 0.336.CC and PSC usually co-occur and are graded using retro-illumination images shown in Figure 14. Retro-illumination images are usually in pairs as each lens has two images of it, one focusing on the anterior cortex (anterior image, Figure 14 top row) and the other, 3–5 mm posterior to it, close to the posterior capsule (posterior image, Figure 14 bottom row). Most of the CC is present in the anterior cortex, and so it is sharply visible in anterior image. On the other hand, PSC is clearer in posterior image as compared to the anterior image.

Typical features used for grading CC and PSC include enhanced texture features [56], intensity, edge, size and spatial location based features [285], entropy [57] and Symlets wavelet coefficients and intensity features [58]. In [58] the grading accuracy is represented in terms of correlation of the predicted grades with the actual and the value of correlation coefficient is 0.7392.

##### ***Corneal opacity***

Corneal haze describes the condition when the cornea becomes cloudy or opaque. The cornea is normally clear, so corneal haze can greatly impair vision. Although the haze can occur in any part of the cornea, it is most often found within the thicker, middle layer of the cornea, called the stroma. Corneal haze is most often caused by inflammatory cells and other debris that are activated during trauma, infection or surgery. Corneal haze sometimes occurs during laser vision correction procedures.

Slit lamp imaging has been used to clinically estimate corneal haze manually by physicians but not automatically. For example, it was used to observe the cornea haze after excimer laser ablation of cornea [286,287]. Slit lamp suffers from resolution reduction caused by interference of light reflected from structures above and below the plane of examination. Confocal microscopy uses a condenser to focus the light source within a small area of the cornea and an objective, which has the same focal point (hence the term confocal) as the condenser. Therefore it is possible to avoid light contamination from out-of-focal information. Compared with slit lamp, an advantage of confocal microscope is a much higher spatial resolution. Moreover, it allows real-time viewing of structures in the living cornea at the cellular level in four dimensions (x, y, z, and time). It can be used to measure corneal haze [288,289].

The above imaging modalities have been used in clinic with manual detection but till now, as far as we know, there is no automatic method based on these modalities. Currently, the existing automatic method is based on the most straightforward way: examining frontal photograph of eye [290,291]. In [291], five situations are considered: cataract, iridocyclitis, corneal haze, corneal arcus, and normal eyes. In the proposed method, each image is first preprocessed using histogram equalization and K-means clustering. The extracted features are then fed into a RBF based neural network classifier.

## Abbreviations

AMD: Age-related macular degeneration; AM-FM: Amplitude modulation-frequency modulation; BM: Bruch’s membrane; BMO: Bruch’s membrane opening; CAD: Computer aided detection; CASNET: Causal association network; CC: Cortical cataract; CCT: Central corneal thickness; CDR: Cup-to-disc ratio; CDSS: Clinical decision support systems; CME: Cystoid macular edema; CNV: Choroidal neovascularization; CSLO: Confocal scanning laser ophthalmoscopy; DH: Disc haemorrhage; DR: Diabetic retinopathy; FBIF: Focal biologically inspired feature; GRI: Glaucoma risk index; GWAS: Genome-wide association studies; HALT: Histogram based adaptive local thresholding; HE: Hard exudates; HRT: Heidelberg retina tomography; ICA: Independent component analysis; IOP: Intra-ocular pressure; LASSO: Least absolute shrinkage selector operation; LDA: Linear discriminant analysis; MGG: Mixture of generalized Gaussian; MLP: Multilayer perceptron; MOG: Mixture of Gaussian; MRA: Moorfields regression analysis; MRI: Magnetic resonance imaging; MTA: Major temporal arcade; NC: Nuclear cataract; NCO: Neural canal opening; NRR: Neuro-retinal rim; OCT: Optical coherence tomography; OMIM: Online Mendelian inheritance in man; PCA: Principal component analysis; PM: Pathological myopia; PPA: Parapapillary atrophy; PSC: Posterior sub-capsular; QDA: Quadratic discriminant analysis; RNFL: Retina nerve fibre layer; RNFLT: Retina nerve fibre layer thickness; ROI: Region of interest; RPE: Retinal pigment epithelium; SAP: Standard automated perimetry; SD-OCT: Spectral domain-optical coherence tomography; SEAD: Symptomatic exudate-associated derangements; SIFT: Scale-invariant feature transform; SLIC: Simple linear iterative clustering; SLP: Scanning laser polarimetry; SNP: Single-nucleotide polymorphism; SNR: Signal to noise ratio; SS-OCT: Swept source-optical coherence tomography; SVDD: Support vector data description; SVM: Support vector machines; TCA: Topographic change analysis; VCD: Vertical cup diameter; VDD: Vertical disc diameter.

## Competing interests

The authors declare that they have no competing interests.

## Authors’ contributions

ZZ conceived topic of this survey, did the review for ocular genomic data, and image related PM research and wrote the concerned parts of the manuscript. RS surveyed and wrote on Cataract and DR and consolidated the final manuscript. HL surveyed and wrote on the topics of AMD detection and corneal opacity detection. XC surveyed and wrote about Glaucoma screening and also enriched the section on CAD of ocular diseases based on clinical data. LD clarified the motivation of this survey and wrote the conclusion. JL, CKK and DWKW conceived of the survey and participated in designing it. TYW provided clinical advice. All authors read and approved the final manuscript.

## Pre-publication history

The pre-publication history for this paper can be accessed here:

http://www.biomedcentral.com/1472-6947/14/80/prepub
